# The phosphite oxidoreductase gene, *ptxD as* a bio-contained chloroplast marker and crop-protection tool for algal biotechnology using *Chlamydomonas*

**DOI:** 10.1007/s00253-019-10258-7

**Published:** 2019-12-02

**Authors:** Saowalak Changko, Priscilla D. Rajakumar, Rosanna E. B. Young, Saul Purton

**Affiliations:** Algal Research Group, Institute of Structural and Molecular Biology, University College London, Gower Street, London, WC1E 6BT UK

**Keywords:** Bio-containment, *Chlamydomonas*, Chloroplast, Contamination, *ptxD*, Phosphite, Oral vaccine, Selectable marker

## Abstract

Edible microalgae have potential as low-cost cell factories for the production and oral delivery of recombinant proteins such as vaccines, anti-bacterials and gut-active enzymes that are beneficial to farmed animals including livestock, poultry and fish. However, a major economic and technical problem associated with large-scale cultivation of microalgae, even in closed photobioreactors, is invasion by contaminating microorganisms. Avoiding this requires costly media sterilisation, aseptic techniques during set-up and implementation of ‘crop-protection’ strategies during cultivation. Here, we report a strain improvement approach in which the chloroplast of *Chlamydomonas reinhardtii* is engineered to allow oxidation of phosphite to its bio-available form: phosphate. We have designed a synthetic version of the bacterial gene (*ptxD*)-encoding phosphite oxidoreductase such that it is highly expressed in the chloroplast but has a Trp→Opal codon reassignment for bio-containment of the transgene. Under mixotrophic conditions, the growth rate of the engineered alga is unaffected when phosphate is replaced with phosphite in the medium. Furthermore, under non-sterile conditions, growth of contaminating microorganisms is severely impeded in phosphite medium. This, therefore, offers the possibility of producing algal biomass under non-sterile conditions. The *ptxD* gene can also serve as a dominant marker for genetic engineering of any *C. reinhardtii* strain, thereby avoiding the use of antibiotic resistance genes as markers and allowing the ‘retro-fitting’ of existing engineered strains. As a proof of concept, we demonstrate the application of our *ptxD* technology to a strain expressing a subunit vaccine targeting a major viral pathogen of farmed fish.

**Electronic supplementary material:**

The online version of this article (10.1007/s00253-019-10258-7) contains supplementary material, which is available to authorized users.

## Introduction

The microalgal chloroplast is an attractive industrial biotechnology platform for synthesis of recombinant products such as bioactive metabolites and therapeutic proteins (Gimpel et al. 2015a; Taunt et al. 2017). Of the handful of microalgal species for which transformation of the chloroplast genome has been reported, the most advanced is the freshwater chlorophyte *Chlamydomonas reinhardtii* (Purton et al. 2013). This model species has been used to demonstrate the successful synthesis of numerous therapeutic proteins in the chloroplast (Dyo and Purton 2018) and is now being explored as a platform for industrial enzymes, RNA-based vaccines and complex metabolites such as terpenoids (Yan et al. 2016; Charoonnart et al. 2019; Zedler et al. 2015). The advantages of the microalgal chloroplast as a cell factory include: (i) the capability to grow the biomass in closed photobioreactor systems using light energy and a minimal medium (Gimpel et al. 2015b); (ii) the ability of the chloroplast to serve as a sub-cellular compartment that accumulates recombinant product without perturbing the metabolism of the rest of the algal cell (Tran et al. 2013); (iii) the simple genetic system that lends itself well to synthetic biology strategies in which transgenes can be fused to highly active *cis* elements and targeted to precise loci within the chloroplast genome (‘plastome’), allowing predictive high-level expression without issues of gene silencing (Boehm and Bock 2019; Dyo and Purton 2018).

One of the major economic and technical challenges to commercial production of recombinant products in microalgae is avoiding contamination and culture collapse in photobioreactors (PBRs) through opportunistic invasion by bacteria, fungi, other algae, or protozoa (Day et al. 2012; Wang et al. 2013). Not only does this necessitate the costly sterilisation of large volumes of media and the aseptic set-up of the PBRs (Hines et al. 2010), but given that most PBR systems are relatively low tech compared with modern industrial fermenters (Gupta et al. 2015), then keeping the algal culture free of major contaminants during operation is also a challenge. This may require the addition of expensive antibiotics and other biocides or inhibitory chemicals that target the main invading species whilst having minimal effect on the growth of the algae (Wang et al. 2013). An alternative ‘crop-protection’ strategy is to use extremophile algae that are adapted to grow under conditions of high salinity (e.g. *Dunaliella salina*) or low pH and high temperature (e.g. *Cyanidioschyzon merolae*) thereby favouring growth of the algae over the most contaminating species. Although chloroplast engineering (transplastomics) has been reported recently for both these species, the molecular tools are poorly developed and *C. reinhardtii* remains the preferred platform for microalgal transplastomics (Dyo and Purton 2018; Scranton et al. 2015).

Recently, Loera-Quezada et al. (2016) described a simple protection strategy for microalgae that builds on pioneering work aimed at controlling weeds during cultivation of crop plants (López-Arredondo and Herrera-Estrella 2012). This approach exploits the fact that plants and algae can actively import phosphite (Phi: HPO_3_^2−^) from the soil or media but are unable to use it as a source of phosphorus: rather, normal growth is dependent on an exogenous supply of phosphate (Pi: PO_4_^3−^) (López-Arredondo and Herrera-Estrella 2012; Loera-Quezada et al. 2015). This inability of Phi to serve as a bio-available form of phosphorus appears to hold for all eukaryotes and most prokaryotes, with only a few bacterial groups shown to possess a metabolic pathway for selective uptake of Phi and its oxidation to Pi (Loera-Quezada et al. 2015, Achary et al. 2017). The best characterised pathway is that of *Pseudomonas stutzeri* WM88, with the key enzyme being PtxD: a phosphite oxidoreductase that utilises NAD+ to oxidise Phi to Pi (Metcalf and Wolfe 1998). The creation of transgenic *Arabidopsis* and tobacco lines expressing *ptxD* (López-Arredondo and Herrera-Estrella 2012), demonstrated that plants could be engineered to utilise Phi and thereby out-compete weeds when grown using a Phi-based fertiliser. Subsequent studies have extended this *ptxD*-engineering approach to important crops such as cotton (Manna et al. 2016) and rice (Pandeya et al. 2018), as well as to industrial microorganisms such as *Escherichia coli* and yeasts (Shaw et al. 2016; Motomura et al. 2018) giving them a selective advantage over contaminating microorganisms when cultured in Phi-based media. Similarly, Loera-Quezada et al. (2016) demonstrated that expression of *ptxD* in the nucleus of *C. reinhardtii* resulted in transgenic lines able to grow in a medium containing Phi as the sole source of phosphorus, and that these strains had a strong selective advantage over contaminating or competing species.

A further application of *ptxD* is as a dominant selectable marker for genetic engineering of prokaryotic or eukaryote species, whereby transformant lines are selected directly for their ability to grow on Phi-containing medium. Such a metabolic marker is superior to the frequently used antibiotic resistance markers since selection uses a cheap substrate (Phi) and eliminates the problem of ‘false positives’ since the spontaneous acquisition of Phi metabolism is not possible. Furthermore, the use of metabolic markers helps address the regulatory and safety concerns associated with the environmental spread of antibiotic resistance genes by horizontal gene transfer (HGT) during commercial cultivation (EFSA GMO Panel 2004; Beacham et al. 2017). Various reports have shown that *ptxD* can serve as an efficient marker for generation of transgenic plants (López-Arredondo and Herrera-Estrella 2013; Nahampun et al. 2016; Pandeya et al. 2017), yeasts (Kanda et al. 2014) and cyanobacteria (Selão et al. 2019). However, whilst the very low abundance of Phi in the natural environment means that HGT of the *ptxD* marker to other microbial species is unlikely to confer any selective advantage, this could still compromise its use as for crop protection. For example, fungal pathogens of plants or microbial competitors of yeast, bacterial or microalgal platforms could acquire *ptxD* and then thrive under Phi-cultivation conditions (Hirota et al. 2017).

With this in mind, we have sought to develop a bio-contained version of *ptxD* as a selectable marker for engineering the *C. reinhardtii* chloroplast and as a crop-protection tool that circumvents the need for media sterilisation. Using a simple codon reassignment strategy, we developed a synthetic version of *ptxD* that contains two internal stop codons. Correct translation therefore requires a chloroplast-specific synthetic tRNA capable of reading the stop codons as tryptophan codons (Young and Purton 2016). Consequently, any escape of the *ptxD* DNA to other microorganisms is very unlikely to give rise to a functional gene. We have demonstrated that expression of *ptxD* in the *C. reinhardtii* chloroplast results in significant levels of the enzyme and allows the engineered strains to grow as effectively on Phi as on Pi under mixotrophic conditions. We have shown that *ptxD* can be used directly for selection and have defined a neutral integration site within the chloroplast genome, allowing us to ‘retrofit’ a previous chloroplast transformant expressing a fish vaccine. Importantly, we have demonstrated that large-scale cultivation of this new strain is feasible in non-sterile media with minimal growth of contaminants, even when such contaminants are deliberately inoculated into the photobioreactor. This work further supports the practical application of *ptxD* as both a benign marker and as a crop-protection system. In principle, our *ptxD* variant could be introduced into any existing strain of *C. reinhardtii* including those engineered to synthesise novel products such as recombinant proteins, biofuels and novel metabolites. During the completion of this work, the group of Badillo-Corona also reported the successful expression of a separate version of *ptxD* in the *C. reinhardtii* chloroplast (Sandoval-Vargas et al. 2018, 2019).

## Materials and methods

### Algal strains and culture conditions

The genotypes of *C. reinhardtii* strains used in this work are detailed in Table S1, with stocks maintained on 2% agar plates containing Tris-acetate phosphate (TAP) medium (Harris et al. 2009) under dim light (5–10 μE m^−2^ s^−1^) at 20 °C. Indirect selection of chloroplast transformants carrying *ptxD* was carried out using the non-photosynthetic mutant TN72 (CC-5168: *cw15*, *psbH*::*aadA*, mt+) with selection for restoration of photosynthetic ability on agar plates containing high salt minimal (HSM) medium and incubated under 50 μE m^−2^ s^−1^ light at 25 °C, as described previously (Wannathong et al. 2016). Direct selection for *ptxD* transformants was on agar plates containing Tris-acetate medium supplemented with sodium phosphite (Na_2_HPO_3_·5H_2_O, 04283 Sigma-Aldrich) at a final concentration of 1 mM (referred to as TA-Phi medium: Table S2). Liquid cultures were grown in either TAP or TA-Phi media in conical flasks under 50 μE m^−2^ s^−1^ light at 25 °C, shaking at 120 rpm. TA-Phi plates were prepared using molecular-grade agar (ThermoFisher Scientific, BP1423-500) to avoid issues with trace phosphate contamination. Cell concentration in liquid cultures was determined by measuring optical density at 750 nm using a spectrophotometer. Comparative growth experiments were carried out in 1 l flasks under controlled conditions of lighting, temperature and aeration using a dual chamber Algem photobioreactor (Algenuity, UK) with the optical density measured in situ at set times during the cultivation.

### Plasmid construction

To generate a plasmid-carrying *ptxD*, the synthetic gene was codon-optimised for the chloroplast of *C. reinhardtii* (Fig. S1). The gene was designed with two TGG→TGA codon alterations and synthesised by GeneArt (ThermoFisher Scientific). *Sap*I and *Sph*I sites were placed immediately upstream and downstream, respectively, of the coding sequence to allow cloning into chloroplast expression vector pWUCA2 (Young and Purton 2016. See: www.chlamycollection.org for sequence details). Plasmid pWUCA2-ptxD was propagated in *E. coli* DH5α and extracted using a QIAfilter Plasmid Midi kit (Qiagen, Venlo, The Netherlands). To create a *ptxD* plasmid for the retro-fitting experiment, the whole gene cassette (i.e. *ptxD* fused to the *psaA-1* promoter/5′UTR and the *rbcL* 3′UTR) was amplified from pWUCA2-ptxD by PCR such that an *Mlu*I site and an *Mfe*I site were located at the upstream and downstream ends, respectively (see Table S3 for primer details). This was then cloned into the *Mlu*I and *Eco*RI sites of plasmid pBa3-AX plasmid (Hallahan et al. 1995) such that the *ptxD* cassette was located in a neutral region between *psaA-3* and *trnL2*, as illustrated in Fig. 5a. The resulting plasmid was named as pBa3-AX-ptxD. Finally, plasmid pPO3 was created by cloning the *trnW*^UCA^ gene into the *Mlu*I site of pBa3-AX-ptxD (Figs. S2 and S3).

### Chloroplast transformation in *C. reinhardtii*

Chloroplast transformation was performed using the vortex method in which a cell suspension is agitated in the presence of the plasmid and glass beads, followed by plating on selective media (Wannathong et al. 2016). For direct selection on phosphite medium, cultures were grown to a density of 2 × 10^6^ cells ml^−1^ in TAP, harvested by centrifugation at 4000×*g*, and the cell pellet washed with TA-Phi medium to remove trace phosphate and resuspended in TA-Phi to a final concentration of 2 × 10^8^ cells ml^−1^. Following agitation with glass beads and plasmid, cells were plated onto TA-Phi 2% agar plate using molten TA-Phi 0.5% agar (Wannathong et al. 2016). Plates were incubated at 25 °C under 50 μE m^−2^ s^−1^ white light for 2–3 weeks. To achieve homoplasmy (in which all plastome copies carry the engineered change), transformant lines generated using *psbH* as the selectable marker were restreaked to single colonies two times under selection (i.e. on HSM plates). Transformants generated using *ptxD* as a marker were taken through four rounds of single-colony isolation on TA-Phi plates. Integration of the transgene and homoplasmy was confirmed by PCR analysis of genomic DNA extracted from a single colony (Werner and Mergenhagen 1998). Primers used for PCR analysis are given in Table S3.

### Western blot analysis

For Western blot analysis, protein samples of mid-log phase *C. reinhardtii* at equal cell density were prepared and separated by SDS-PAGE using a gel containing 15% acrylamide as described in Young and Purton (2014). The proteins were blotted onto a Hybond ECL nitrocellulose membrane (GE Healthcare) using a Trans-Blot SD semi-dry electrophoretic transfer cell (Bio-Rad) at 19 V for 1 h. The membrane was blocked overnight in TBS-T (TBS + 0.1% Tween) with 5% milk and incubated with a primary antibody (α-HA antibody produced in rabbit diluted at 1:2000, Sigma-Aldrich product H6908) for 1 h. After washing the membrane in TBS-T for 30 min (5–15 min each time), it was incubated with a secondary antibody (Goat anti-rabbit IgG, DyLight 800 diluted at 1:25,000, Thermo Scientific product 35571) for 1 h and followed again by washing in TBS-T. Both antibodies were diluted in TBS-T with 0.5% milk. For detection, the membrane was analysed using the Odyssey Infrared Imaging system (Li-COR Biosciences).

### Functional analysis of *ptxD* transgenic lines

Growth on solid medium was tested by culturing strains in TAP followed by transfer to TA medium (i.e. without any phosphate) for 3 days to deplete the cells of internal stores of polyphosphate (Komine et al. 2000). Cell samples were prepared to equal optical density measured at 750 nm by resuspension in TA medium, and 5 μl spotted onto either TAP or TA-Phi solid medium. Plates were incubated under 50 μE m^−2^ s^−1^ light at 25 °C for 4 days. To determine growth rates in liquid media, a stationary-phase inoculum of 25 ml previously grown in TA-Phi was seeded into a flask containing 400 ml of either TAP or TA-Phi and grown in an Algem Labscale Photobioreactor (Algenuity, UK). Growth was determined by measurement in situ of the optical density of the cultures at 740 nm.

### Large-scale cultivation of transgenic lines in a ‘hanging bag’ photobioreactor

Scale-up performance was assessed in a hanging bag photobioreactor system originally developed by the Cawthron Institute, New Zealand (Taunt et al. 2017). The NNV + PtxD strain was grown in single-use polythene tubular bags (provided by Supreme Health, New Zealand), each containing 20 l of TAP or TA-Phi medium. An inoculum of 500 ml was grown to stationary phase in TA-Phi under standard growth conditions. Each bag was then seeded with 500 ml inoculum, together with 1 ml of a contaminant stock of natural bacteria and fungi that was generated by allowing a flask of TAP medium to become deliberately contaminated by leaving it open to the air in the lab for several days prior to incubation at 25 °C. The hanging bags were sparged from the bottom with filter-sterilised air and illuminated at 100 μE m^−2^ s^−1^ using Osram Lumilux Cool daylight fluorescence tubes. Cultures were cultivated for 3 days at 25 °C and then the algal/bacterial populations assessed by particle size distribution using a Mastersizer 3000 laser diffraction particle size analyser (Malvern Panalytical Ltd., UK).

#### Sequence data

The Genbank accession number of the synthetic *ptxD* used in the study is MK492115.

## Results

### Generation of chloroplast transformants containing a ‘bio-contained’ *ptxD*

**Fig. 1:**
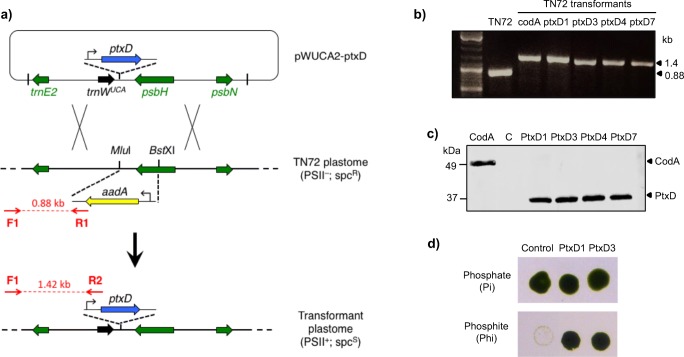
Generation and characterisation of *ptxD* transformants. **a** The pWUCA2-ptxD plasmid contains *ptxD* under the control of the *psaA* exon 1 promoter/5′UTR and *rbcL* 3′UTR. The *trnW*_*UCA*_ gene immediately upstream allows translational readthrough of the two TGA stop codons in *ptxD* (Young and Purton 2016). Targeted integration of both genes into the *psbH-trnE2* intergenic region of the plastome of the *psbH* mutant TN72 occurs via two homologous recombination events, resulting in replacement of the *aadA* cassette and restoration of photosynthetic function. **b** PCR confirmation of plastome integration using three primers per reaction. As depicted in (**a**), the original TN72 plastome yields a 0.88 kb product with primers F1 and R1, whereas the transformant plastome gives a 1.42 kb product with primers F1 and R2. The absence of a 0.88 kb band for all four transformants indicates that the plastomes are homoplasmic, as seen for the control transformant (codA). **c** Western blot analysis of PtxD protein accumulation in the transgenic lines. An anti-HA antibody was used to detect the HA-tagged PtxD protein (37 kDa) in crude cell lysates. The codA transformant expressing an HA-tagged cytosine deaminase protein (49 kDa) serves as a positive control, whilst a TN72 transformant generated using the empty pWUCA2 plasmid serves as a negative control. An equivalent amount of cell lysate was loaded in each lane. **d** Growth tests of two transformant lines and the negative control on solid Tris-acetate medium containing either phosphate or phosphite. Plates were photographed 4 days after spotting of equivalent volumes of culture onto each plate

A synthetic version of the *ptxD* gene from *Pseudomonas stutzeri* WM88 was designed by optimising the codon usage for efficient translation in the *C. reinhardtii* chloroplast. The coding sequence was extended at the 3′ end to include sequence for a haemagglutinin (HA) epitope tag at the C-terminus of the protein. Finally, our bio-containment feature was built into the gene design whereby two of the tryptophan codons (UGG) were changed to UGA stop codons. The synthetic gene was then cloned into the chloroplast expression plasmid pWUCA2 such that it was placed under the control of the chloroplast *psaA-1* promoter and 5′UTR and the *rbcL* 3′UTR (Young and Purton 2016). The plasmid also carries a copy of the chloroplast tRNA^Trp^ gene in which the anticodon has been modified to recognise UGA, and has flanking elements to target both genes into a neutral region of the plastome downstream of *psbH* via homologous recombination (Fig. 1a). Transformant colonies were recovered following glass bead-mediated transformation of the non-photosynthetic *ΔpsbH* mutant, TN72 such that selection of transformant colonies was based on rescue of TN72 to phototrophy (Wannathong et al. 2016).

Four phototrophic colonies were chosen at random, restreaked on minimal medium and then checked by PCR analysis for integration of *ptxD* into the plastome. As shown in Fig. 1b, all four lines gave rise to the 1.4-kb band expected if the cassette integrated into the target locus downstream of *psbH*, whereas the untransformed TN72 gave rise to a band of 0.88 kb. Furthermore, the absence of any detectable 0.88 kb band from the transformants indicated that the lines are homoplasmic with no copies of the original TN72 plastome remaining in the chloroplast. To check for expression of *ptxD*, western analysis was carried out using antibodies to the HA epitope tag. The calculated molecular weight of the HA-tagged PtxD is 37 kDa, and analysis of crude cell lysates confirmed the accumulation of the full-length recombinant protein in all the transgenic lines, as shown in Fig. 1c. Importantly, the production of the full-length PtxD protein in the chloroplast demonstrates that translational read-through of the two internal stop codons does occur, as previously shown for the *codA* gene (see Fig. 1c and Young and Purton (2016)) and for the *nnv* gene (see below).

### The PtxD protein allows robust growth of transgenic lines in medium containing phosphite as the sole source of phosphorus

To examine whether the PtxD protein is functional and capable of converting sufficient phosphite to phosphate for growth, cultures of two of the transformant lines and a control transformant lacking *ptxD*, were spotted onto solid medium containing either phosphate or phosphite at 1 mM. As shown in Fig. 1d, all three grew well on the phosphate medium, but only the *ptxD* transformants were capable of equivalent growth on phosphite, demonstrating that the PtxD is active in the algal chloroplast and able to support robust growth under the selective conditions.

**Fig. 2:**
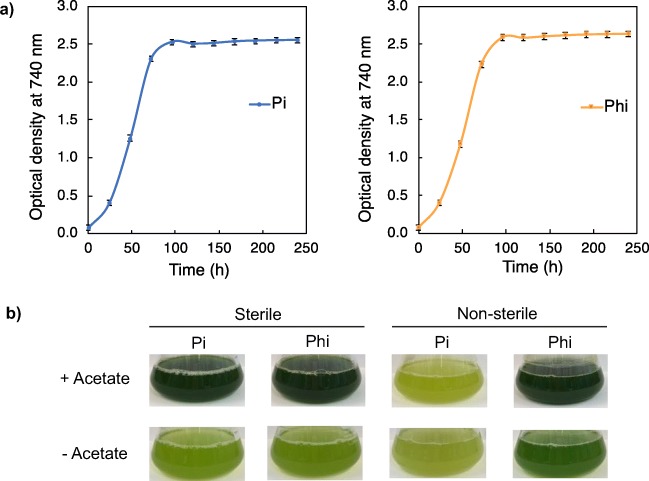
Growth of transgenic line PtxD1 in medium supplemented with phosphate (Pi) or phosphite (Phi). **a** A P-depleted culture was used as an inoculum and growth carried out in Algem photobioreactors under equivalent conditions of light, temperature and mixing for 10 days. Optical density of each culture was measured at 740 nm. Each curve is derived from two biological replicates with error bars representing standard deviation**. b** In a separate experiment, PtxD1 cultures were grown under sterile and non-sterile conditions in phosphite and phosphate media with or without acetate as a fixed carbon source. The mixotrophic cultures (+acetate) were growth to stationary phase over a period of 7 days, whereas the phototrophic cultures (–acetate) required 10 days owing to the slower growth of *C. reinhardtii* in phototrophic mode (Chapman et al. 2015)

A more detailed comparison of the phosphate versus phosphite growth kinetics was then carried out using PtxD1 as the representative strain. PtxD1 was grown in duplicate in either liquid TAP or TA-Phi media under identical conditions of temperature lighting and aeration using Algem photobioreactors. As shown in Fig. 2a, an essentially identical profile was obtained for the two sources of phosphorus, indicating that under these conditions the transport of phosphite across the cell and chloroplast membranes, and its oxidation to phosphate and subsequent distribution within the cell are not rate limiting.

### Use of phosphite allows selective growth of the PtxD transformant in contaminated media

Loera-Quezada et al. (2016) previously demonstrated that nuclear expression of *ptxD* in *C. reinhardtii* allows efficient cultivation in phosphite media under non-sterile conditions, with the engineered strain outcompeting microbial contaminants. We therefore examined whether expression of *ptxD* in the chloroplast similarly provides such contamination protection. Strain PtxD1 was grown in liquid culture in media containing either Pi or Phi under both mixotrophic (acetate present in the medium) and phototrophic (no acetate) conditions. In one experiment, the media were sterilised; whereas in the parallel experiment, the media were not sterilised and were deliberately contaminated at the start of the cultivation using a cocktail of natural bacteria and fungi that had been allowed to grow in TAP medium. As shown in Fig. 2b, healthy growth of the strain is seen under sterile conditions, with no obvious differences in cell density at stationary phase when grown in either Pi or Phi media, although phototrophic growth results in a lower cell density as has been well established (e.g. Chapman et al. 2015). In contrast, there is a marked difference in appearance between the Pi and Phi cultures when grown under the non-sterile conditions. In Phi media, the growth is comparable with that achieved under sterile conditions, whereas there is a marked reduction in algal growth under both phototrophic and mixotrophic conditions when the media contains Pi as an available source of phosphorus for the contaminants. Microscopic examination of such cultures showed extensive bacterial contamination, whilst much less contamination was seen in Phi cultures (Fig. S4), further supporting the idea that the use of phosphite media can serve as an effective crop-protection strategy.

**Fig. 3:**
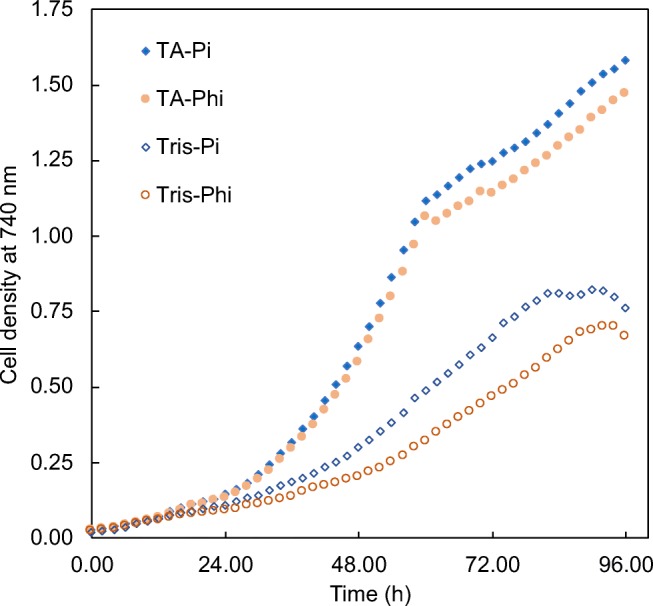
Growth of transgenic line PtxD1 under phototrophic (‘Tris’ medium) or mixotrophic (‘Tris-acetate’ (TA) medium) conditions with either phosphate (Pi) or phosphite (Phi) as the phosphorus source. The four media were inoculated from a culture of cells grown in TAP medium and grown over 96 h in the Algem photobioreactors with growth rates monitored at 740 nm over 96 h under identical conditions of temperature, lighting and mixing

One potential issue with employing PtxD in the chloroplast is that conversion of Phi to Pi requires oxidising equivalents in the form of NAD+ (or less efficiently, NADP+) (Costas et al. 2001). Whilst mixotrophic growth of *C. reinhardtii* allows the supply of oxidising equivalents to the chloroplast from the mitochondrion as a consequence of oxidative phosphorylation (Johnson and Alric 2013), under phototrophic conditions the NAD+/NADP+ pool in the chloroplast is significantly more reduced as the light reactions of photosynthesis drive the conversion of NADP+ to NADPH by ferredoxin: NADP(H) oxidoreductase (Goss and Hanke 2014). Phototrophic growth of a PtxD transformant could therefore result in a very small pool of NAD+/NADP+ and hence low Phi-to-Pi conversion by a chloroplast-localised PtxD, leading to reduced growth in Phi medium owing to Pi limitation. We explored this hypothesis by comparing the growth rates of PtxD1 under these two phototrophic conditions: i.e. where PtxD activity is required (Phi medium) and where PtxD activity is not required (Pi medium). As shown in Fig. 3, a marked reduction in the growth rate is seen when the transformant strain is required to grow phototrophically and convert Phi into a usable source of phosphorus. In comparison, growth under mixotrophic conditions—which is also significantly higher overall because of the availability of acetate as a reduced carbon source (Chapman et al. 2015)—is not affected by the substitution of Pi for Phi during the exponential growth phase (see also Fig. 2a). It is only when the acetate becomes depleted towards the end of this phase and there is a switch to phototrophic growth, do we observe reduced growth on Phi.

### *ptxD* as a non-antibiotic selectable marker for chloroplast transformation

**Fig. 4:**
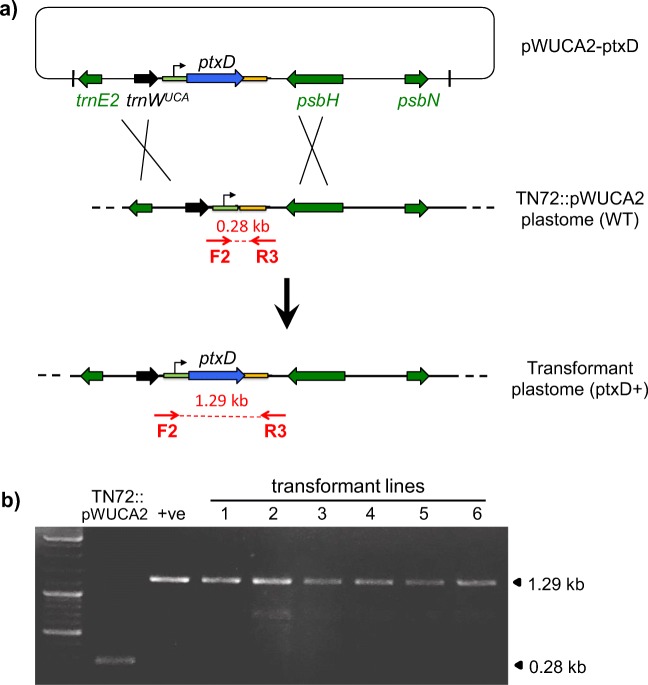
Use of *ptxD* as a selectable marker for direct selection of transformants on phosphite medium. **a** Schematic diagram showing transformation of the control strain TN72::pWUCA2 (wildtype phenotype) with plasmid pWUCA2-ptxD such that a double recombination event introduces *ptxD* downstream of *psbH*. **b** Confirmation of *ptxD* integration in six independent transformant lines by PCR using primers F2 and R3 as shown the diagram. Prior to integration, the PCR product is 0.28 kb as shown for TN72::pWUCA2 whereas successful integration gives an equivalent transgene arrangement as for strain PtxD1 (positive control) with a PCR product of 1.29 kb

The use of *ptxD* a selectable marker for genetic engineering is attractive as it circumvents the risk and regulatory issues associated with antibiotic-based markers and their possible horizontal transfer to microbial pathogens. Furthermore, the ability to metabolise Phi to Pi is very unlikely to arise spontaneously in the recipient cell so false positives are unlikely during transformation. Given that there is a dearth of selectable markers for engineering the algal chloroplast (Day and Goldschmidt-Clermont 2011; Esland et al. 2018), we tested whether transformation of *C. reinhardtii* using pWUCA2-ptxD could be achieved by direct selection on Tris-acetate medium containing 1 mM Phi as the only source of phosphorus. As an initial test, we used the negative control strain (TN72 transformed to phototrophy using pWUCA2) as a recipient line, as illustrated in Fig. 4a. Following glass bead-mediated transformation, the colonies became visible within two weeks, with the green lawn of non-transformant cells dying back. No colonies were seen on the ‘no-plasmid DNA’ control plates. Six colonies were selected, restreaked once on fresh TA-Phi plates and checked by PCR for integration of *ptxD*. As shown in Fig. 4b, all six lines contain the gene and appear to be homoplasmic.

**Fig. 5:**
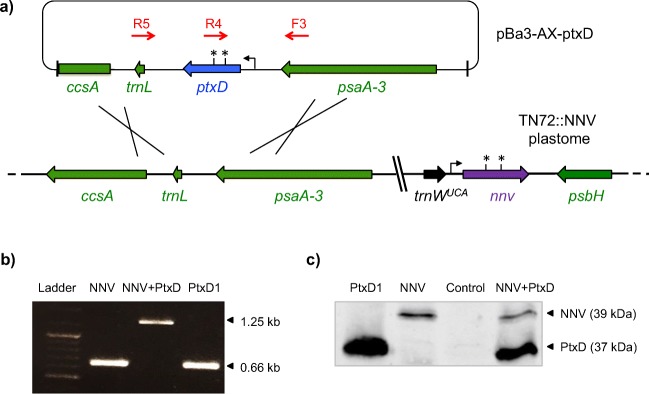
‘Retrofitting’ a chloroplast transformant line for phosphite metabolism. **a** Targeting of the *ptxD* marker into a neutral locus on the plastome of the TN72::NNV strain by transformation with plasmid pBa3-AX-ptxD and direct selection on phosphite. TN72::NNV was previously engineered to express the *nnv* gene encoding the capsid protein from the fish pathogen, nervous necrosis virus. Note that both *nnv* and *ptxD* contain internal TGA stop codons (asterisk) that are read as tryptophan by the tRNA encoded by the introduced *trnW*^UCA^ gene. **b** ‘Three-primer’ PCR analysis of a representative transformant (NNV + PtxD) to confirm the successful insertion of *ptxD.* Primers F3 and R4 give an expected band of 1.25 kb, whereas the original NNV line gives a band of 0.66 kb with F3 and R5, as does the PtxD1 transformant. **c** Western blot analysis of the NNV + PtxD transformant using anti-HA antibodies to show the accumulation of both HA-tagged proteins: the NNV capsid and PtxD at approximately 39 and 37 kDa, respectively. TN72 transformed with the empty pWUCA2 plasmid was used as a negative control

To test whether the whole *ptxD* expression cassette (together with the *trnW*^UCA^ gene required for *ptxD* translation) could be used as a portable dominant marker, we examined whether it could be targeted to another neutral site in the plastome between *psaA-3* and *trnL*. This would allow us to introduce phosphite metabolism capability into any *C. reinhardtii* strain including existing chloroplast transformants generated using TN72. Two experiments were therefore carried out. In the first, we sought to transform a TN72 line previously engineered to produce a subunit vaccine against the fish pathogen Nervous Necrosis Virus (NNV) (Rajakumar 2016). The *ptxD* cassette was cloned into the *psaA-3-trnL* intergenic region on plasmid pBa3-AX as shown in Fig. 5a, although *trnW*^UCA^ was not included in the plasmid construct as the NNV plastome already carried this gene for translational readthrough of TGA stop codons in the *nnv* gene. Glass-bead-mediated transformation yielded several colonies on phosphite medium. Analysis of a representative transformant confirmed that *ptxD* had integrated into the plastome and that the resulting line produced both the NNV and PtxD proteins (Fig. 5b, c).

For more general applications, we created a modified plasmid named pPO3 in which *trnW*^UCA^ is also carried on pBa3-AX-ptxD (Fig. S2). This plasmid could be used for conversion to phosphite metabolism of any nuclear- or chloroplast-engineered strain of *C. reinhardtii* or indeed any strain domesticated through mutagenesis or sexual crosses (e.g. Fields et al. 2019). Since most strains possess a wild-type cell wall, and therefore would not be suitable for glass-bead-mediated transformation (Wannathong et al. 2016), we tested whether pPO3 could be used for direct selection of transformants on phosphite medium using the microparticle bombardment method. As similarly reported by Sandoval-Vargas et al. (2019), *ptxD* transformant colonies were readily recovered following bombardment of a wild-type strain (CC-1690). The integration of the *ptxD* cassette into the plastome between *psaA-3* and *trnL*, and the homoplasmy of the transformants was confirmed by PCR as shown in Fig. S5.

### Large-scale cultivation under non-sterile conditions illustrates the utility of the phosphite system

**Fig. 6:**
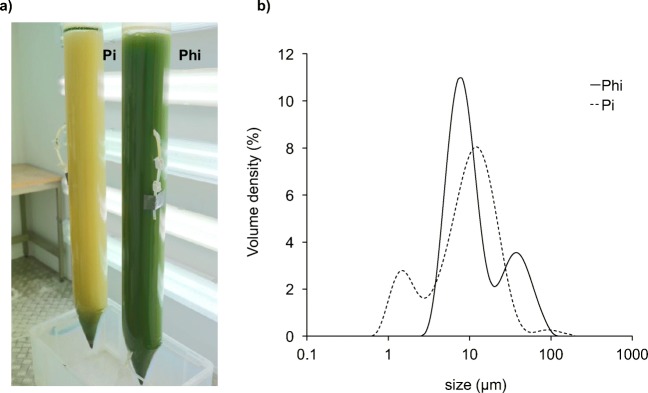
Large-scale cultivation of the NNV + PtxD strain in a ‘hanging bag’ system under non-sterile conditions. **a** Photograph of the cultures grown using non-sterile phosphate (Pi) and phosphite (Phi) media after 13 days. Each 20 l bag is inoculated with a starter culture (2.5%, *v*/*v* inoculum) and a culture of natural contaminants (0.005%, *v*/*v*) and grown mixotrophically with ~ 100 μE illumination and bubbling with sterile air at 2.5 l min^−1^. **b** Estimation of contamination by Mastersizer analysis, showing a distibution of bacterial contaminants and *C. reinhardtii* cells at peak of approximately 1–3 and 10 μm, respectively

The NNV transformant is part of an on-going ‘proof-of-concept’ project as a cheap, oral vaccine for the farmed fish industry (Charoonnart et al. 2018). However, production of algal biomass for applications such as feed additives for the aquaculture, poultry and other livestock sectors, can be challenging, not least because of the problems of contamination in low-cost photobioreactor systems (Taunt et al. 2017). To demonstrate the practical use of the *ptxD* system as a crop-protection tool, we carried out a large-scale cultivation of the NNV + PtxD transgenic line in a hanging-bag system. The Tris-acetate medium supplemented with Pi or Phi was prepared without sterilisation, and in order to demonstrate a random chance of contamination by bacteria, we also added a small volume of TAP medium that had naturally become contaminated. As shown in Fig. 6a, the Pi culture was a bleached yellow color after 13 days of cultivation, with few viable algal cells (as observed by microscopy, Fig. S6). In contrast, the Phi-treated cells remained a healthy green colour with minimal observable contamination. The level of contamination in the two cultures was estimated by particle size analysis (Fig. 6b). A large peak of *Chlamydomonas*-sized cells (~ 10 μm diameter, plus clusters of cells around ~ 50 μm) is seen in the Phi culture. In contrast, the level is lower in the Pi culture with the increased size suggesting that the cells are stressed. What is most obvious in the Pi culture is the peak of bacterial-sized cells (1–3 μm) which is not seen in the Phi culture.

## Discussion

Whilst there is considerable interest in the integration of microalgae such as *Chlamydomonas reinhardtii* into industrial biotechnology—and much has been written regarding their potential as low-cost, light-driven cell factories for making valuable recombinant proteins and metabolites (e.g. Gimpel et al. 2015a, Lauersen 2019, Taunt et al. 2017)—the economic and technical challenges of crop protection when cultivating microalgae at scale are often overlooked. Even in ‘closed’ systems such as photobioreactors or internally lit fermentors, the potential for spoilage through contamination with bacteria, fungi or other algae is high, especially if operated over an extended time period or in continuous mode (Day et al. 2012; Wang et al. 2013). Current crop-protection strategies typically involve an expensive practice of using biocides and antibiotics to kill contaminating species (Bacellar Mendes and Vermelho 2013). Alternatively, extreme conditions of pH, salinity or temperature are used to favor the growth of the algae over these contaminants. However, this strategy is only possible for a few extremophilic species such as the halophile *Dunaliella salina* or the acidophilic Cyanidiales (Varshney et al. 2015).

The PtxD/phosphite system offers a simple and economic crop-protection strategy for industrial cultivation of *C. reinhardtii* (Loera-Quezada et al. 2016; Sandoval-Vargas et al. 2018). This versatile freshwater species is currently the most genetically tractable microalga with established molecular tools for engineering both the nuclear and chloroplast genomes (Scaife et al. 2015; Crozet et al. 2018). As such, *C. reinhardtii* has been proposed as a cell factory for a wide range of products including pharmaceutical proteins (Dyo and Purton 2018; Yan et al. 2016), RNA-based vaccines for aquaculture (Somchai et al. 2016; Charoonnart et al. 2019), dietary enzymes for livestock (Erpel et al. 2016; Manuell et al. 2007), novel isoprenoids as bioactives (Lauersen 2019), biopolymers (Chaogang et al. 2010) and specialist biofuels such as biohydrogen and bisabolene (Torzillo et al. 2015; Wichmann et al. 2018). All of these engineered strains could be ‘retrofitted’ using our *ptxD* cassette to allow selective growth on phosphite media, thereby avoiding the production costs associated with sterilising the media and protecting the algal crop from spoilage. This is particularly useful where the product is of relatively low value, such as a biofuel, biopolymer or commodity chemical, and therefore economic production demands large-scale, non-sterile cultivation in low-tech PBR systems or in open-pond systems (Chacón-Lee and González-Mariño 2010; Resurreccion et al. 2012). As we have demonstrated here, production of a *C. reinhardtii* strain to be used as an oral vaccine for fish can be achieved in a simple hanging bag PBR system using the phosphite strategy without any sterility control measures.

By expressing *ptxD* within the algal chloroplast rather than the nucleus (as previously achieved by Loera-Quezada et al. 2016), we were able to incorporate an effective bio-containment feature into the gene. Here, two TGA stop codons were inserted within the coding region, such that full-length translation requires the co-expression in the chloroplast of a modified tRNA gene (Young and Purton 2016). Since chloroplast tRNAs are very unlikely to be functional in bacteria (or indeed in other eukaryotic protists such as fungi and oomycetes), any horizontal transfer of *ptxD* to these microorganisms is not likely to give rise to phosphite metabolism. This ‘functional bio-containment’ feature therefore helps address one of the major issues with any crop-protection strategy: namely the emergence of novel contaminants able to survive and compete under the selective conditions.

We have also demonstrated that our *ptxD* cassette can serve as a dominant selectable marker for chloroplast transformation and have constructed suitable plasmids for targeting the cassette into a neutral site on the plastome (either at the *psbH-trnE2* or the *psaA-3-trnL* locus). Sandoval-Vargas et al. (2019) have also recently reported the use of *ptxD* as a selectable marker for the *C. reinhardtii* chloroplast, and our combined work adds a useful dominant marker to the molecular toolbox (Esland et al. 2018). Importantly, this marker is not derived from a bacterial antibiotic resistance gene and therefore raises less concerns regarding GM regulation (EFSA GMO Panel 2004; Beacham et al. 2017). Finally, selection of transformants based on phosphite metabolism does not suffer from false-positive issues as spontaneous mutants able to oxidise phosphite are very unlikely to arise.

In theory, *ptxD* could be used as a selectable marker for chloroplast engineering of other microalgal species, and for introduction of phosphite metabolism for industrially important strains. Chloroplast transformation has been reported for several key species that are grown commercially, including *Haematococcus pluvialis*, *Dunaliella tertiolecta*, *Nannochloropsis oceanica*, *Phaeodactylum tricornutum* and *Euglena gracilis* (see: Esland et al. 2018). However, there are two important considerations. The first is whether the native phosphate transporters of these organisms are able to import phosphite both into the cell and into the chloroplast. Early work by Yehudai-Resheff et al. (2007) indicated that phosphite can be transported to the chloroplast of *C. reinhardtii*, and this was comfirmed by the work of Sandoval-Vargas et al. (2018). However, recent studies of cyanobacteria transformed with *ptxD* have revealed species-specific differences. Whereas a transformant of *Synechococcus* sp. PCC 7002 carrying just the *ptxD* transgene was capable of growth on phosphite (Selão et al. 2019), growth of *Synechococcus elongatus* PCC 7942 or *Synechocystis* sp. PCC 6803 transformants required additional transgenes encoding the cognate phosphite transporter (Motomura et al. 2018, Polyviou et al. 2015). Since chloroplasts have evolved from cyanobacteria through endosymbiosis, it is possible that such differences in natural phosphite uptake exist in the different algal species, especially those (e.g. *Nannochloropsis* and *Euglena*) whose chloroplasts have been acquired by secondary endosymbiosis (Keeling 2013).

The second consideration is that use of the PtxD/phosphite system in algal cultivation may negatively impact phototrophic growth by depleting reducing equivalents, as we have proposed here for *C. reinhardtii*. For obligate phototrophs (e.g. *Haematococcus pluvialis*), this might limit the utility of the system as a crop-protection strategy. However, for *C. reinhardtii*, this appears not to be a concern since this species can use acetate as a fixed carbon source, and our studies show that under these conditions growth rates on phosphite appear unaffected. Because of the much higher productivities achieved when *C. reinhardtii* is fed acetate (Chapman et al. 2015), then mixotrophic growth in photobioreactors (or heterotrophic growth in fermentors) is likely to be the approach for commercial production of most recombinants in this microalgal platform (Fields et al. 2018). Ironically, the addition of a carbon source such as acetate to the culture medium increases the risk of contamination by heterotrophic microbes making the PtxD/phosphite system even more valuable as a crop-protection tool.

## Electronic supplementary material

ESM 1 (PDF 15990 kb)
