# Supplementary material for: The phosphite oxidoreductase gene, ptxD as a bio-contained chloroplast marker and crop-protection tool for algal biotechnology using Chlamydomonas
Source: Appl Microbiol Biotechnol. 2019 Dec 2;104(2):675–86. doi: 10.1007/s00253-019-10258-7 (PMC6943410; doi:10.1007/s00253-019-10258-7)
Supplement: Supplementary file 1 — (PDF 15990 kb) [file 253_2019_10258_MOESM1_ESM.pdf]

<u>Electronic Supplementary Material</u>

The bacterial phosphite oxidoreductase gene, *ptxD* as a bio-contained chloroplast marker and crop protection tool for microalgal biotechnology using *Chlamydomonas*

Saowalak Changko, Priscilla D. Rajakumar, Rosanna E. B. Young and Saul Purton

Algal Research Group, Institute of Structural and Molecular Biology, University College London, Gower Street, London, WC1E 6BT, United Kingdom

Table S1 *Chlamydomonas* strains used

Table S2 Composition of phosphite medium, TA-Phi

Table S3 Primers used in plasmid construction and confirmation of homoplasmic integration in chloroplast transformants

Figure S1 Coding sequence and protein sequence of codon-optimized *ptxD* gene for the *C. reinhardtii* chloroplast

Figure S2 Plasmid map of pPO3

Figure S3 DNA sequence of plasmid pPO3

Figure S4 Microscopy examination of contamination levels following growth of PtxD1 in non-sterile Tris-acetate medium containing either phosphate or phosphite.

Figure S5 PCR analysis to confirm recovery and homoplasmy of *ptxD* transformants following biolistic transformation of wild-type (WT) strain CC-1690 with plasmid pPO3

Figure S6 Microscopic observation following large-scale cultivation of PtxD+NNV strain in phosphate and phosphite medium

Table S1 *Chlamydomonas* strains used

Strain name and phenotype	Culture collection code (www.chlamy.com)	Comments
**TN72**<br>PSII-minus; cell-wall deficient	CC-5168	Non-photosynthetic recipient (*psbH*::*aadA*) used for indirect selection of *ptxD* transformants by glass bead-mediated transformation.
**Wild-type**	CC-1690	Used to demonstrate direct selection with *ptxD* by biolistic transformation.
**TN72::pWUCA2**<br>cell-wall deficient	N/A	TN72 transformed with empty pWUCA2 vector used as a recipient for the direct selection of *ptxD* transformants by glass bead-mediated transformation.

Table S2 Composition of phosphite medium, TA-Phi (i.e. Tris-acetate medium supplemented with phosphite as the sole source of phosphorus).

Component	Concentration
NH_4Cl	0.40 g/L
$MgSO_4 \cdot 7H_2O$	0.05 g/L
$CaCl_2 \cdot 2H_2O$	0.10 g/L
$Na_2HPO_3 \cdot 5H_2O$, 1 M, pH 7.0	1.00 mM
KCl, 1 M, pH 7.0	1.00 mM
Tris	2.42 g/L
Glacial acetic acid, 100%	1.00 ml/L
Trace elements stock solution<br>(see: Kropat et al. 2011)	7.00 ml/L

Table S3 Primers used in plasmid construction and confirmation of integration of transgenes into the plastome

**Primer**	**Sequence 5′-3′**	**Comments**
Primers used in plasmid construction		
ptxD.F	AGGTTTCGGATTGAACAATAATGGC	Amplification of *ptxD* gene for cloning into pBa3-AX. Note, the *Mlu*I site is located downstream of the forward primer; the *Mfe*I site was introduced into the reverse primer (changes shown in lowercase).
ptxD.R	CCACTAcaaTTgCGTTAAGATAAACGC GCTACATC	
Primers used to confirm integration and homoplasmy (shown in Fig.1a, 5a and 6a).		
F1	GTCATTGCGAAAATACTGGTGC	To confirm the integration of *ptxD* into the plastome. The primer binds to flanking sequence located outside of the transforming pWUCA2 plasmid, therefore, the amplification is successful only if the transgene integrates at the correct plastome locus.
R1	CGGATGTAACTCAATCGGTAG	R1 combines with F1 to generate a PCR product of 880 bp from the untransformed plastome of the TN72 recipient.
R2	ATAGGCTCTTCTCATGGATTTCTCCTT ATAATAAC	R2 combines with F1 to generate a PCR product of 1415 bp from the plastome of TN72 transformants.
F2	AACTATTTGTCTAATTTAATAACC	F2+R3 used to confirm introduction of *ptxD* into recipient TN72::pWUCA2 following direct selection. PCR products are 282 bp prior to integration, 1286 bp after integration.
R3	CAAACTTCACATGCAGCAGC	See above.
F3	TGGTCGTGGTTACTGGCAAGAA	To confirm the integration of *ptxD* at a neutral site between *psaA-3* and *trnL.* F3 binds toward the end of *psaA-3* in both recipient strain and transformant.
R4	CCCAACCTTGTAAACGGTCAGC	R4 is located within *ptxD* and combines with F3 to generate a PCR product of 1251 bp if the cassette integrates at the locus (1532 bp if the cassette includes *trnW*^{UCA}, as for plasmid pPO3).
R5	CGTCTACCATTCCGCCATATC	R5 is located within *trnL* and combines with F3 to generate a PCR product of 657 bp for the WT locus.

>*ptxD* (1047 bp)

ATGTTACCAAAATTAGTAATTACTCACCGTGTTCACGACGAAATTTTACAATTATTAGCTCC
ACACTGTGAATTAATGACAAACCAAACTGATTCTACTTTAACACGTGAAGAAATTTTACGTC
GTTGTCGTGACGCACAAGCTATGATGGCTTTCATGCCAGATCGTGTTGATGCTGATTTCTTA
CAAGCTTGTCCAGAATTACGTGTAGTTGGTTGTGCTTTAAAAGGTTTCGATAACTTCGATGT
TGATGCTTGTACTGCTCGTGGTGTATGATTAACATTCGTACCAGATTTATTAACAGTACCAA
CAGCTGAATTAGCTATTGGTTTAGCTGTAGGTTTAGGTCGTCACTTACGTGCTGCTGATGCT
TTCGTACGTTCAGGTGAATTCCAAGGTTGACAACCACAATTCTATGGTACTGGTTTAGATAA
CGCTACTGTAGGTATTTTAGGTATGGGTGCTATTGGTTTAGCTATGGCTGACCGTTTACAAG
GTTGGGGTGCTACTTTACAATATCACGAAGCTAAAGCTTTAGATACTCAAACAGAACAACGT
TTAGGTTTACGTCAAGTAGCTTGTTCAGAATTATTTGCTTCTTCTGATTTCATTTTATTAGC
TTTACCATTAAACGCTGATACTCAACACTTAGTAAACGCTGAATTATTAGCTTTAGTTCGTC
CAGGTGCTTTATTAGTTAACCCATGTCGTGGTTCAGTAGTAGATGAAGCTGCTGTATTAGCT
GCTTTAGAACGTGGTCAATTAGGTGGTTACGCTGCTGATGTTTTTGAAATGGAAGATTGGGC
TCGTGCTGACCGTCCACGTTTAATTGACCCTGCTTTATTAGCTCACCCAAACACTTTATTTA
CACCACACATTGGTTCAGCTGTTCGTGCTGTTCGTTTAGAAATTGAACGTTGTGCTGCTCAA
AACATTATTCAAGTATTAGCTGGTGCTCGTCCAATTAACGCTGCTAACCGTTTACCAAAAGC
TGAACCTGCTGCTTGTGGTTCTTACCCATACGATGTTCCAGATTACGCTTAATAA

>NAD:Phosphite oxidoreductase (37.28 kDa)

MLPKLVITHRVHDEILQLLAPHCELMTNQTDSTLTREEILRRCRDAQAMMAFMPDRVDADFL
QACPELRVVGCALKGFDNFDVDACTARGVWLTFVPDLLTVPTAELAIGLAVGLGRHLRAADA
FVRSGEFQGWQPQFYGTGLDNATVGILGMGAIGLAMADRLQGWGATLQYHEAKALDTQTEQR
LGLRQVACSELFASSDFILLALPLNADTQHLVNAELLALVRPGALLVNPCRGSVVDEAAVLA
ALERGQLGGYAADVFEMEDWARADRPRLIDPALLAHPNTLFTPHIGSAVRAVRLEIERCAAQ
NIIQVLAGARPINAANRLPKAEPAACGSYPYDVPDYA*

Figure S1 Coding sequence and protein sequence of codon-optimized *ptxD* gene for the *C. reinhardtii* chloroplast. The gene contains two TGA internal stop codons, highlighted in green, which are translated as tryptophan by the modified tRNA in the engineered algal strains (Young & Purton 2016)

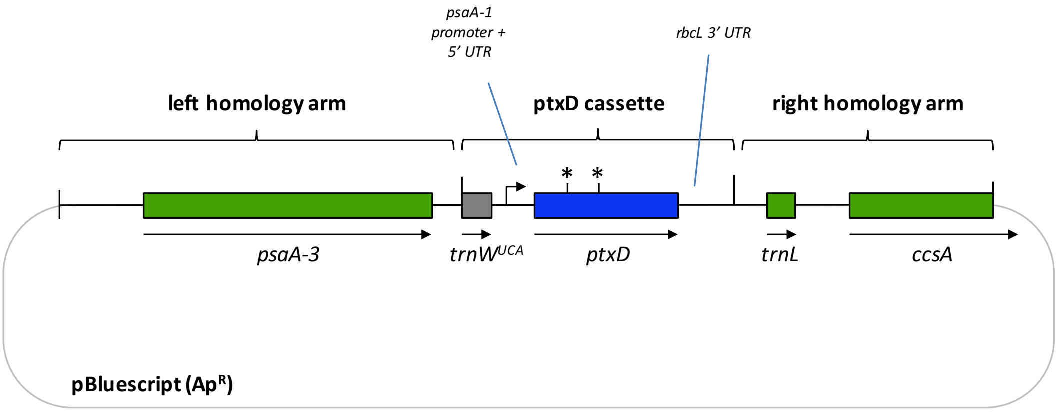

pPO3

ptxD integration plasmid
(9356 bp)

Figure S2 Plasmid pPO3, an integration plasmid for inserting the bio-contained *ptxD* into a neutral location on the *C. reinhardtii* plastome between *psaA-3* and *trnL*. Shown is a schematic diagram of the plasmid highlighting the left and right arms (2.9 kb and 1.5 kb, respectively) flanking the 2.0 kb 'ptxD cassette' that comprises the two parts of the bio-containment system – the codon-optimised *ptxD* containing two internal TGA stop codons (*), and a chloroplast-specific tRNA gene modified to recognize the stop codon as a tryptophan codon

Plasmid pPO3 is available through the Chlamydomonas Resource Center (www.chlamycollection.org).

Figure S3 DNA sequence of plasmid pPO3

>pPO3 (9356 bp)

CCCATCAAGCTTATCGATACCGTCGACCTCGAGGGGGGGCCCGGTACCCAATTCGCCCTATAGTGAGT
CGTATTACAATTCACTGGCCGTCGTTTTACAACGTCGTGACTGGGAAAACCCTGGCGTTACCCAACTT
AATCGCCTTGCAGCACATCCCCCTTTCGCCAGCTGGCGTAATAGCGAAGAGGCCCGCACCGATCGCCC
TTCCCAACAGTTGCGCAGCCTGAATGGCGAATGGAAATTGTAAGCGTTAATATTTTGTTAAAATTCGC
GTTAAATTTTTGTTAAATCAGCTCATTTTTTAACCAATAGGCCGAAATCGGCAAAATCCCTTATAAAT
CAAAAGAATAGACCGAGATAGGGTTGAGTGTTGTTCCAGTTTGGAACAAGAGTCCACTATTAAAGAAC
GTGGACTCCAACGTCAAAGGGCGAAAAACCGTCTATCAGGGCGATGGCCCACTACGTGAACCATCACC
CTAATCAAGTTTTTTGGGGTCGAGGTGCCGTAAAGCACTAAATCGGAACCCTAAAGGGAGCCCCCGAT
TTAGAGCTTGACGGGGAAAGCCGGCGAACGTGGCGAGAAAGGAAGGGAAGAAAGCGAAAGGAGCGGGC
GCTAGGGCGCTGGCAAGTGTAGCGGTCACGCTGCGCGTAACCACCACACCCGCCGCGCTTAATGCGCC
GCTACAGGGCGCGTCAGGTGGCACTTTTCGGGGAAATGTGCGCGGAACCCCTATTTGTTTATTTTTCT
AAATACATTCAAATATGTATCCGCTCATGAGACAATAACCCTGATAAATGCTTCAATAATATTGAAAA
AGGAAGAGTATGAGTATTCAACATTTCCGTGTCGCCCTTATTCCCTTTTTTGCGGCATTTTGCCTTCC
TGTTTTTGCTCACCCAGAAACGCTGGTGAAAGTAAAAGATGCTGAAGATCAGTTGGGTGCACGAGTGG
GTTACATCGAACTGGATCTCAACAGCGGTAAGATCCTTGAGAGTTTTCGCCCCGAAGAACGTTTTCCA
ATGATGAGCACTTTTAAAGTTCTGCTATGTGGCGCGGTATTATCCCGTATTGACGCCGGGCAAGAGCA
ACTCGGTCGCCGCATACACTATTCTCAGAATGACTTGGTTGAGTACTCACCAGTCACAGAAAAGCATC
TTACGGATGGCATGACAGTAAGAGAATTATGCAGTGCTGCCATAACCATGAGTGATAACACTGCGGCC
AACTTACTTCTGACAACGATCGGAGGACCGAAGGAGCTAACCGCTTTTTTGCACAACATGGGGGATCA
TGTAACTCGCCTTGATCGTTGGGAACCGGAGCTGAATGAAGCCATACCAAACGACGAGCGTGACACCA
CGATGCCTGTAGCAATGGCAACAACGTTGCGCAAACTATTAACTGGCGAACTACTTACTCTAGCTTCC
CGGCAACAATTAATAGACTGGATGGAGGCGGATAAAGTTGCAGGACCACTTCTGCGCTCGGCCCTTCC
GGCTGGCTGGTTTATTGCTGATAAATCTGGAGCCGGTGAGCGTGGGTCTCGCGGTATCATTGCAGCAC
TGGGGCCAGATGGTAAGCCCTCCCGTATCGTAGTTATCTACACGACGGGGAGTCAGGCAACTATGGAT
GAACGAAATAGACAGATCGCTGAGATAGGTGCCTCACTGATTAAGCATTGGTAACTGTCAGACCAAGT
TTACTCATATATACTTTAGATTGATTTAAAACTTCATTTTTAATTTAAAAGGATCTAGGTGAAGATCC
TTTTTGATAATCTCATGACCAAAATCCCTTAACGTGAGTTTTCGTTCCACTGAGCGTCAGACCCCGTA
GAAAAGATCAAAGGATCTTCTTGAGATCCTTTTTTTCTGCGCGTAATCTGCTGCTTGCAAACAAAAAA
ACCACCGCTACCAGCGGTGGTTTGTTTGCCGGATCAAGAGCTACCAACTCTTTTTCCGAAGGTAACTG
GCTTCAGCAGAGCGCAGATACCAAATACTGTCCTTCTAGTGTAGCCGTAGTTAGGCCACCACTTCAAG
AACTCTGTAGCACCGCCTACATACCTCGCTCTGCTAATCCTGTTACCAGTGGCTGCTGCCAGTGGCGA
TAAGTCGTGTCTTACCGGGTTGGACTCAAGACGATAGTTACCGGATAAGGCGCAGCGGTCGGGCTGAA
CGGGGGGTTCGTGCACACAGCCCAGCTTGGAGCGAACGACCTACACCGAACTGAGATACCTACAGCGT
GAGCTATGAGAAAGCGCCACGCTTCCCGAAGGGAGAAAGGCGGACAGGTATCCGGTAAGCGGCAGGGT
CGGAACAGGAGAGCGCACGAGGGAGCTTCCAGGGGGAAACGCCTGGTATCTTTATAGTCCTGTCGGGT
TTCGCCACCTCTGACTTGAGCGTCGATTTTTGTGATGCTCGTCAGGGGGGCGGAGCCTATGGAAAAAC
GCCAGCAACGCGGCCTTTTTACGGTTCCTGGCCTTTTGCTGGCCTTTTGCTCACATGTTCTTTCCTGC
GTTATCCCCTGATTCTGTGGATAACCGTATTACCGCCTTTGAGTGAGCTGATACCGCTCGCCGCAGCC
GAACGACCGAGCGCAGCGAGTCAGTGAGCGAGGAAGCGGAAGAGCGCCCAATACGCAAACCGCCTCTC
CCCGCGCGTTGGCCGATTCATTAATGCAGCTGGCACGACAGGTTTCCCGACTGGAAAGCGGGCAGTGA
GCGCAACGCAATTAATGTGAGTTAGCTCACTCATTAGGCACCCCAGGCTTTACACTTTATGCTTCCGG
CTCGTATGTTGTGTGGAATTGTGAGCGGATAACAATTTCACACAGGAAACAGCTATGACCATGATTAC
GCCAAGCTCGAAATTAACCCTCACTAAAGGGAACAAAAGCTGGAGCTCCACCGCGGTGGCGGCCGCTC
TAGGTATATACATTCACCCTTTAAGGCTACCCGGCAGTTAGTTACGGCTTACGTTCCATAAAATATTG
GCATATTTTATAAATTATTTTATAGATCATATATTTTGTAAATATATAATATTACGCATAACACATTT
ATTTAAAAACATATAATATTACGCATAACACATTTATTTAAAAACAGCAAAAACTTGCGTCAAATCCC
TATAGGATATTTTTTATTTATGGCACGAGCAGGTTTACACGCTCCGTCAGGACGCCGGCACGTAGTTG
GAAAGTATGTCCCCCTTGCCCGGAAGGGGAAAGGAGGAGACAAATTTATTTATTGTATATAAATAGCA
TGACTTTCCAAGCGAGTTAACATAAACAAACTGCGCGAGTTAACATAAACAAACTTCCTCTCTGGGGA
GGCATGGCAGCAAATGGCACCCTAAATACATGCGCTTTTAACACAGATAATTATAAACAAGCATAGCG
TTAAATCTGCTACCTTGGATTAAATCATTAGAAAGAATTTGAGCCGTGTGCAGTGAAAATTGCATGCA
CGGCTCTTAAGGTTTAAATAAATTTTTAAAGAAGAAAATTTAACTCCTAACTATGTACTTCCATGGTG

CACGTTTTTCAAACTATGAAGCTTGGTTAAGTGACCCTACTCACATTAAACCAAGTGCTCAAGTAGTA
TGGCCTATTGTAGGTCAAGAAATTTTAAACGGTGATGTAGGTGGTGGTTTCCAAGGTATTCAAATTAC
TTCTGGTTTCTTCCAATTATGGCGTGCTAGTGGTATTACTAGTGAATTACAACTTTATACTACAGCAA
TTGGTGGTTTAGTAATGGCTGCTGCAATGTTCTTTGCTGGTTGGTTCCACTACCACAAAGCTGCTCCA
AAACTAGAATGGTTCCAAAACGTTGAATCAATGTTAAACCACCACTTAGGTGGTCTTCTTGGTTTAGG
TAGTTTAGCTTGGGCTGGTCACCAAATTCACGTTTCTTTACCAGTAAACAAATTATTAGATGCTGGTG
TAGATCCAAAAGAAATTCCACTTCCTCATGATTTATTATTAAATCGTGCTATTATGGCTGACTTATAC
CCAAGTTTTGCTAAAGGTATTGCTCCTTTCTTTACTTTAAACTGGAGTGAATACAGTGATTTCTTAAC
ATTTAAAGGTGGTTTAAACCCTGTTACTGGTGGTCTTTGGTTAAGTGATACTGCTCACCACCACGTAG
CTATTGCTGTATTATTCTTAGTAGCTGGTCACATGTATCGTACTAACTGGGGTATTGGTCACAGTATG
AAAGAAATTTTAGAAGCTCACCGTGGTCCATTTACAGGTGAAGGTCACGTTGGTTTATATGAAATTTT
AACAACTTCTTGGCATGCACAATTAGCTATTAACTTAGCTTTATTTGGTTCGTTATCAATTATTGTAG
CTCACCACATGTACGCAATGCCTCCATACCCTTATTTAGCTACTGATTACGGTACACAATTATCATTA
TTTACACACCACACATGGATTGGTGGTTTCTGTATTGTTGGTGCTGGTGCTCACGCAGCTATTTTCAT
GGTTCGTGACTACGATCCTACTAATAACTACAACAACTTATTAGACCGTGTAATTCGTCACCGTGATG
CTATTATTTCTCACTTAAACTGGGTTTGTATTTTCTTAGGTTTCCACAGCTTTGGTTTATACATCCAC
AACGATACAATGAGTGCTTTAGGTCGTCCTCAAGACATGTTCTCAGATACTGCTATCCAACTTCAACC
AGTATTTGCTCAATGGATTCAAAATACACACTTCTTAGCTCCACAATTAACAGCACCAAATGCTTTAG
CTGCTACAAGTTTAACTTGGGGTGGTGATTTAGTTGCTGTTGGCGGTAAAGTAGCTATGATGCCTATT
TCTTTAGGTACTTCTGACTTTATGGTTCACCACATTCACGCTTTCACAATTCACGTAACTGTGTTAAT
TCTTCTGAAAGGTGTTTTATTTGCTCGTAGCTCTCGTCTTATCCCAGATAAAGCTAACTTAGGTTTCC
GTTTCCCTTGTGACGGTCCTGGTCGTGGCGGTACTTGTCAGGTTTCTGCTTGGGACCACGTATTCTTA
GGTCTTTTCTGGATGTACAACAGCTTATCAATTGTAATTTTCCACTTCAGCTGGAAGATGCAATCTGA
TGTTTGGGGTACGGTTACAGCTTCTGGTGTTTCTCACATTACTGGTGGTAACTTTGCACAAAGCGCTA
ACACAATCAACGGTTGGTTACGTGACTTCTTATGGGCACAATCATCACAAGTAATCCAATCATACGGT
TCAGCTCTATCTGCTTATGGTTTAATTTTCTTAGGTGCTCACTTCGTATGGGCATTCTCGTTAATGTT
CTTATTCTCTGGTCGTGGTTACTGGCAAGAACTTATCGAATCAATTGTATGGGCTCACAACAAACTTA
AAGTTGCACCTGCAATTCAACCACGTGCTTTAAGTATTACTCAAGGTCGTGCTGTTGGTGTAGCTCAC
TACCTTTTAGGTGGTATTGCTACTACATGGTCGTTCTTCTTAGCACGTATCATTTCTGTAGGTTAACA
TTTAATACTTTTTAATACATATATGCCTAAGTTTATCTTTAAAGATAAACTTAGCCATATGTGTTAAG
TTATCTAACAAGGTTACCTTTTTATTTCTCTTTAGATATATAAACATTAAAAACTACCGTGATCGTTA
CACTTTAGATAACTGGAAGGGGGAAAAATCATGTATTCGCTGGAAGGCGCACCTCCTACTGCCTACTG
CGCAGCATTAAAATGCTGTAGATATTGGTATCTTACAAAGGACAGTAGTACACAATTAAACGCGTTAA
CCCATGATTAACAACTATATCAATAAAATCAATTTGTAGTGAAATACTCTGATTGACATTAAAATAAT
ACCATGATAAAAATTATAATAACAAATTTT**ACGTCCTTAGTTCAGTCGGTAGAACGCAGGTTTTCAAA**
ACCTGATGTCGTGGGTTCAATTCCTACAGGGCGTGTTTTTCCTAATGTACTTTGTTGTAAAAGTGGCT
GGTTTAACCTTTTTAGGTTTCGGATTGAACAATAATGGCAGTTAAGAGTCACTAAAGCTGCTGTATAG
ACGCGTAAGCTTTCTTAATTCAACATTTTTAAGTAAATACTGTTTAATGTTATACTTTTACGAATACA
CATATGGTAAAAAATAAAACAATATCTTTAAAATAAGTAAAAATAATTTGTAAACCAATAAAAAATAT
ATTTATGGTATAATATAACATATGATGTAAAAAAAACTATTTGTCTAATTTAATAACCATGCATTTTT
TATGAACACATAATAATTAAAAGCGTTGCTAATGGTGTAAATAATGTATTTATTAAATTAAATAATTG
TTATTATAAGGAGAAATCCATGTTACCAAAATTAGTAATTACTCACCGTGTTCACGACGAAATTTTAC
AATTATTAGCTCCACACTGTGAATTAATGACAAACCAAACTGATTCTACTTTAACACGTGAAGAAATT
TTACGTCGTTGTCGTGACGCACAAGCTATGATGGCTTTCATGCCAGATCGTGTTGATGCTGATTTCTT
ACAAGCTTGTCCAGAATTACGTGTAGTTGGTTGTGCTTTAAAAGGTTTCGATAACTTCGATGTTGATG
CTTGTACTGCTCGTGGTGTATGATTAACATTCGTACCAGATTTATTAACAGTACCAACAGCTGAATTA
GCTATTGGTTTAGCTGTAGGTTTAGGTCGTCACTTACGTGCTGCTGATGCTTTCGTACGTTCAGGTGA
ATTCCAAGGTTGACAACCACAATTCTATGGTACTGGTTTAGATAACGCTACTGTAGGTATTTTAGGTA
TGGGTGCTATTGGTTTAGCTATGGCTGACCGTTTACAAGGTTGGGGTGCTACTTTACAATATCACGAA
GCTAAAGCTTTAGATACTCAAACAGAACAACGTTTAGGTTTACGTCAAGTAGCTTGTTCAGAATTATT
TGCTTCTTCTGATTTCATTTTATTAGCTTTACCATTAAACGCTGATACTCAACACTTAGTAAACGCTG
AATTATTAGCTTTAGTTCGTCCAGGTGCTTTATTAGTTAACCCATGTCGTGGTTCAGTAGTAGATGAA
GCTGCTGTATTAGCTGCTTTAGAACGTGGTCAATTAGGTGGTTACGCTGCTGATGTTTTTGAAATGGA
AGATTGGGCTCGTGCTGACCGTCCACGTTTAATTGACCCTGCTTTATTAGCTCACCCAAACACTTTAT
TTACACCACACATTGGTTCAGCTGTTCGTGCTGTTCGTTTAGAAATTGAACGTTGTGCTGCTCAAAAC
ATTATTCAAGTATTAGCTGGTGCTCGTCCAATTAACGCTGCTAACCGTTTACCAAAAGCTGAACCTGC
TGCTTGTGGTTCTTACCCATACGATGTTCCAGATTACGCTTAATAAGCATGCAAGCTTGTACTCAAGC

TCGTAACGAAGGTCGTGACCTTGCTCGTGAAGGTGGCGACGTAATTCGTTCAGCTTGTAAATGGTCTC
CAGAACTTGCTGCTGCATGTGAAGTTTGGAAAGAAATTAAATTCGAATTTGATACTATTGACAAACTT
TAATTTTTATTTTTCATGATGTTTATGTGAATAGCATAAACATCGTTTTTATTTTTATGGTGTTTAGG
TTAAATACCTAAACATCATTTTACATTTTTAAAATTAAGTTCTAAAGTTATCTTTTGTTTAAATTTGC
CTGTCTTTATAAATTACGATGTGCCAGAAAAATAAAATCTTAGCTTTTTATTATAGAATTTATCTTTA
TGTATTATATTTTATAAGTTATAATAAAAGAAATAGTAACATACTAAAGCGGATGTAGATGTAGCGCG
TTTATCTTAACGCAATTCCCATATAGCCAATGGCTTAAGGAGTGTCATAGGAATAACTAGTCATGCAC
ATTTTCCTAAAAATCTAAAATGTTATTAGAAGCTATACAAAAATTAAAAATTATGTTATTATATATTT
ATTAAAATTTAGACTTATCACGGGGATATGGCGGAATGGTAGACGCTACGGACTTAAAATCCGTTCTT
GTGCGAACAAGGTGAGGGTTCAAGTCCCTCTTTCCCCATTTATAAATAAAAATCAAGTCAATATTTTA
ACATTGTAAACTAAAAAATAAGTGTAAAATAACTAGGGTAAATAGTTAAGAATTGGTAGTTTTTGAAC
TAAACAGTGACATAAAACTGGTTAAAAGACATCCCTGTAAGAGAAATGCATATGGTGAATTACACAAT
AAATTTTAAAAATAAAGCTCTGACAAGCCTCTTCCCCTTTGCGATATACATGCTCTGTTAGGATATAA
TTCTGCCTAAGTTCCTAACAAAGGGTTTACTTTTTCTCGGGGAATGGTTTTGCTACTTTGTTTTAAAT
CTTTTTCAAAGAATGTCAATCTCCTATAAAGGGAGGAACATTACTCATGGTCTCACAATTTTAATAAA
CTAGTTTCTTATAATCTATTTTATAAAAATAAATCTAAATATATTTCTTATGAATTTTGTTAATTTAG
AACAAATTGAAAATTCTTTACGTAATGCTACTTTTTGCATGCTTTTTTTAACAACATTTTTATATTGG
TTTTATACTGCATTTTACAGTACAAATCCTCAACAAATTATAAATCCATTGTCATTAACAAACATTAA
AACTAATTTTACTTATCCTGACGGGTTTCAAGCAAACAATATTAATGTTTCTACAACTTATTTACCGA
TTAACCCTGTATTAAATACCGAAAGAGAAGAGCAACCCGAAGCTAACGGAACGAATGGGCTGTTGGGT
GTTTCTTCATTAGTAGTAAATTTAAAATCAATAGCAATCCCACGCATTATGATGGGTGTTTCTAATTT
ATTATTAGTATTATTATTACTTGTTCGTTGGGAAAAATCAGGTCATTTCCCATTAAGTAATTTATATG
AATCTTTAATGTTTTTAGCGTGGTGTTGTACATTTTTATATTTATTATATTGTACAAGTTTTACTTTA
TTGGTTGAAAAAATGTTAGGTTCATTAATTGCACCTTGTAGTTTATTAATGAATGCATTTGCTACTTT
TAGTTTACCAAAAGAAATGCAACAAGCATCACCATTAGTACCAGCTTTACAATCAAATTGGTTAATGA
TGCATGTTACTGTAATGATTATTAGTTATGCCACATTAATTATTGGATCGTTATTGTCGATTTTATTT
TTGATTTTATTTAAACACAAAAAAGGTACACCCAAAAAGTATGATAACTTTATTAACAATTTAGATGC
ATTAAGTTATCGCATTATTGGATTAGGTTTTCCTTTTTTAACTATTGGGATTTTATCTGGGGCTGTGT
GGGCTAATGAAGCATGGGGATCATATTGGAGTTGGGATCC

key:

Vector sequence

Coding sequences of chloroplast genes: psaA-3; trnL; ccsA

Element carrying $trnW^{UCA}$ (coding region in **bold**)

ptxD cassette: comprising psaA-1 promoter/5’UTR:ptxD CDS:rbcL 3’ UTR

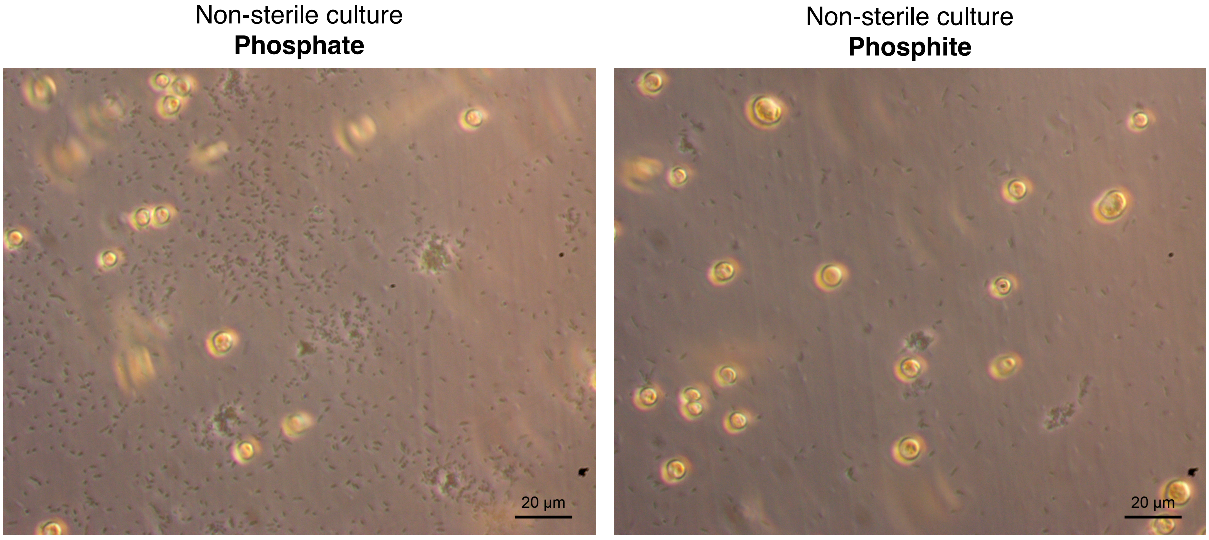

Figure S5 Microscopy examination of contamination levels following growth of PtxD1 in non-sterile and deliberately contaminated Tris-acetate medium containing either phosphate or phosphite. Images were taken after three days' growth in hangings bags [Figure 6a]. Whilst algal cells (some motile) are seen in both images, significantly more bacterial contamination (smaller dark cells) are observed in the phosphate medium.

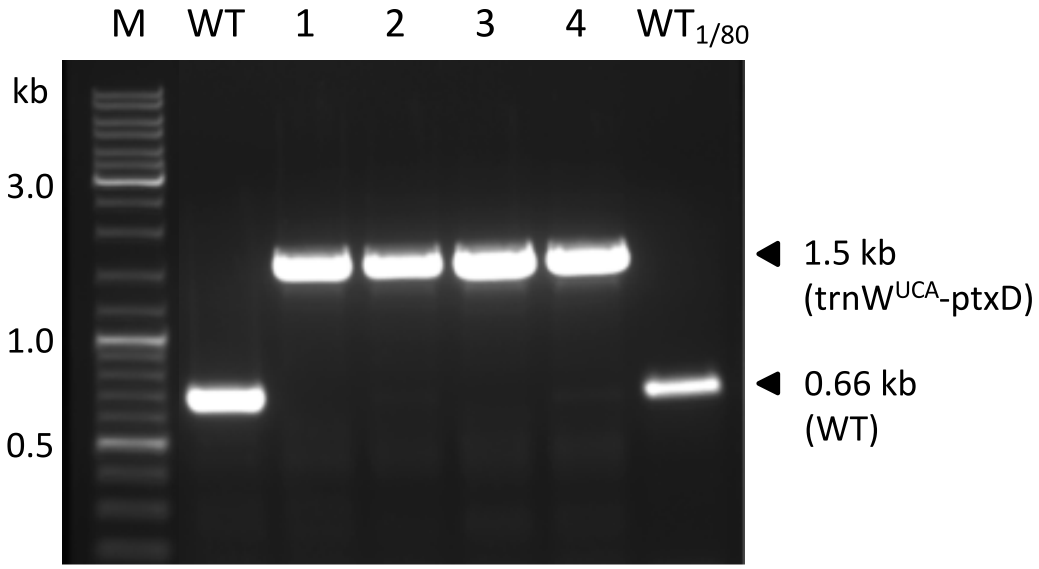

Figure S5 PCR analysis to confirm recovery and homoplasmy of *ptxD* transformants following biolistic transformation of wild-type (WT) strain CC-1690 with plasmid pPO3. PCR analysis was carried out on genomic DNA extracted from four transformant lines following four rounds of streaking out to single colonies on TA-Phi medium. Three primers (F3, R4, R5) were employed, and are predicted to give a 0.66 kb band for the WT control and 1.53 kb for transformant lines due to the integration of the $trnW^{UCA}$ and *ptxD* genes. Since the *C. reinhardtii* plastome is present in ~80 copies per cell (Eberhard et al. 2002), homoplasmy was confirmed by demonstrating that a WT plastome could be detected using this PCR screen even when the WT DNA sample was diluted 80-fold ($WT_{1/80}$) thereby representing the situation of a single copy of the WT plastome remaining in the transformant cell. The four transformants are considered homoplasmic since no 0.66 kb band is detected

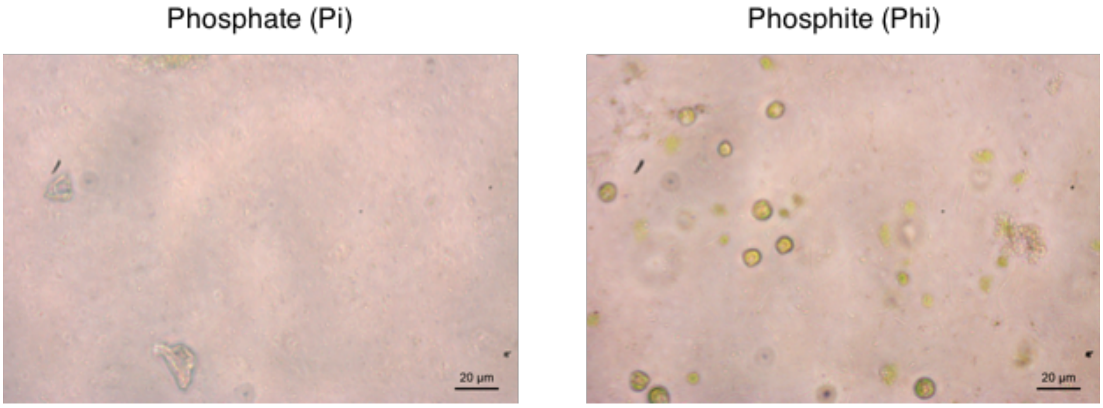

Figure S6 Microscopic examination following large-scale cultivation of PtxD+NNV strain in phosphate and phosphite medium showing a marked reduction in algal cells in the phosphate medium.

Supplementary references:

Eberhard S, Drapier D, Wollman FA (2002) Searching limiting steps in the expression of chloroplast-encoded proteins: relations between gene copy number, transcription, transcript abundance and translation rate in the chloroplast of *Chlamydomonas reinhardtii.* Plant J 31:149–160. https://doi.org/10.1046/j.1365-313X.2002.01340.x

Kropat J, Hong-Hermesdorf A, Casero D, Ent P, Castruita M, Pellegrini M, Merchant SS, Malasarn D (2011). A revised mineral nutrient supplement increases biomass and growth rate in *Chlamydomonas reinhardtii*. Plant J 66:770–780. https://doi.org/10.1111/j.1365-313X.2011.04537.x

Young REB, Purton S (2016) Codon reassignment to facilitate genetic engineering and biocontainment in the chloroplast of *Chlamydomonas reinhardtii*. Plant Biotechnol J 14:1251–1260. https://doi.org/10.1111/pbi.12490
